# FFR-CT: Technical Advances and Implementation in Clinical Practice

**DOI:** 10.3390/jimaging12050202

**Published:** 2026-05-05

**Authors:** Kamil Stankowski, Amedeo Pellizzon, Luca Signorelli, Andrea Baggiano, Nicola Cosentino, Alberico Del Torto, Fabio Fazzari, Daniele Junod, Maria Elisabetta Mancini, Riccardo Maragna, Manuela Muratori, Luigi Tassetti, Alessandra Volpe, Saima Mushtaq, Gianluca Pontone

**Affiliations:** 1 Department of Perioperative Cardiology and Cardiovascular Imaging, Centro Cardiologico Monzino IRCCS, 20138 Milan, Italy; kamil.stankowski@cardiologicomonzino.it (K.S.);; 2 Department of Clinical Sciences and Community Health, University of Milan, 20122 Milan, Italy; 3 Department of Biomedical, Surgical and Dental Sciences, University of Milan, 20122 Milan, Italy

**Keywords:** fractional flow reserve, cardiac computed tomography, coronary artery disease, computational fluid dynamics, machine learning, chronic coronary syndrome

## Abstract

Fractional flow reserve derived from coronary computed tomography angiography (FFR-CT) has emerged as a non-invasive modality for the functional assessment of coronary artery disease. By using computational fluid dynamics, particularly in its most extensively validated off-site implementation, FFR-CT enables lesion-specific estimation of pressure gradients across coronary stenoses without the need for invasive catheterization. This narrative review summarizes the technical foundations of FFR-CT as well as the evidence demonstrating that FFR-CT enhances the diagnostic accuracy of coronary CT angiography alone by improving specificity for hemodynamically significant stenoses when compared with invasive fractional flow reserve. Beyond diagnosis, FFR-CT provides incremental prognostic information, supporting risk stratification and guiding revascularization decisions. Suggestions for clinical implementation of FFR-CT and guidance on interpreting results within the appropriate clinical context are provided. Despite these advantages, limitations remain, including dependence on image quality, reduced performance in heavily calcified vessels, assumptions regarding hyperemic flow conditions, and limited validation in certain populations. While computational fluid dynamics-based FFR-CT remains the most commonly adopted approach in clinical settings, machine learning-based on-site FFR-CT is rapidly evolving and is expected to become a reliable alternative. As technical refinements continue, FFR-CT is poised to play an expanding role in precision-guided management of coronary artery disease.

## 1. Introduction

Coronary artery disease (CAD) stands as a leading cause of morbidity and mortality worldwide. Therefore, a precise diagnostic assessment remains pivotal in identifying patients who could benefit from revascularization strategies [1,2]. Traditionally, the diagnosis and management of CAD have relied on invasive coronary angiography (ICA), which, despite being the gold standard, is associated with procedural risks, resource utilization and high observer disagreement. In this context, cardiac computed tomography (CCT) surges as the preferred diagnostic tool for symptomatic patients with low or intermediate pre-test probability of CAD, as recommended by the 2024 European Society of Cardiology and 2023 American Heart Association/Americal College of Cardiology guidelines for the management of patients with chronic coronary syndromes [3,4]. Integrating CCT into the diagnostic algorithm for patients with stable chest pain significantly decreases cardiovascular death and non-fatal myocardial infarction, compared to standard care [5,6].

Nevertheless, despite the excellent ability of CCT to provide a deep phenotyping of coronary atherosclerosis and its proven diagnostic performance in identifying obstructive stenoses, both of which carry incremental prognostic value [7,8,9,10], it lacks high specificity in identifying functionally significant stenoses [8]. While the probability of being functionally significant increases with the anatomical degree of stenosis, numerous coronary stenoses in the moderate (50–69%) or severe (70–99%) range on CCT fail to show hemodynamic relevance when subjected to invasive fractional flow reserve (FFR) measurement [11,12]. Notably, guidelines underlie that either percutaneous or surgical myocardial revascularization is needed for some patients with functionally significant disease [3,4]. This highlights a relevant limitation inherent to all anatomic imaging techniques, which favor morphological assessment of plaques over their actual functional impact on myocardial perfusion [13].

FFR derived from coronary computed tomography angiography (FFR-CT), a non-invasive assessment of the hemodynamic significance of coronary artery stenoses, represents a significant advancement in this context, as it offers a connection between anatomical and functional evaluation of coronary atherosclerosis [14]. The aim of this review is to expound on a comprehensive evaluation of FFR-CT, with particular emphasis on its technical characteristics, diagnostic and prognostic performances, established clinical applications, and emerging future directions. Given the stronger evidence base, this review will primarily focus on off-site computational flow dynamics (CFD)-based FFR-CT, while also addressing emerging on-site machine learning approaches that are poised to become practical and reliable alternatives. Compared with previous reviews, (i) we examine the use of FFR-CT across specific patient subsets and clinical scenarios, including patients evaluated in the emergency department, those with coronary anatomy abnormalities or microvascular dysfunction, cases involving revascularization planning, and patients undergoing pre-transcatheter aortic valve implantation assessment, where new evidence has recently emerged; (ii) we provide an up-to-date, structured comparison of emerging technologies that enable on-site FFR-CT computation.

## 2. Technical Basis of FFR-CT

Fractional flow reserve, defined as the ratio of distal coronary pressure to aortic pressure (FFR = Pd/Pa) under conditions of maximal hyperemia and measured during ICA, represents the reference standard to evaluate the hemodynamic impact of coronary artery stenoses and guide lesion-specific coronary revascularization decisions [15]. Multiple clinical trials have shown that FFR-guided revascularization reduces unnecessary percutaneous coronary interventions (PCI) and improves patient outcomes [16,17,18]. Based on these and other data, current guidelines on myocardial revascularization assign a Class I, Level of Evidence A recommendation to FFR for the assessment of coronary artery stenoses with a diameter reduction of 50% to 90%, unless non-invasive evidence of ischemia is present [4].

FFR-CT is a non-invasive physiologic modeling method that utilizes standard CCT datasets to computationally simulate coronary blood flow, pressure gradients, and vascular resistance. The most widely adopted, and currently the only U.S. Food and Drug Administration-approved and CE-marked platform, HeartFlow Inc. (Mountain View, CA, USA), applies advanced CFD algorithms processed off-site to generate patient-specific FFR-CT results. FFR-CT does not require pharmacologic agents such as adenosine, nor coronary pressure wires, reducing patient discomfort, procedural risks, and added costs. The technical basis of FFR-CT calculation integrates anatomical data from CCT with physiological principles to simulate coronary hemodynamics non-invasively through computational modeling of blood flow, treating it as a Newtonian fluid described by the Navier–Stokes equations [19].

From a technical standpoint, the process follows five sequential steps. The first entails the creation of patient-specific anatomic models derived from CCT, necessitating high-resolution segmentation of the coronary tree (including both main vessels and branches) to generate a detailed three-dimensional representation of the patient’s coronary anatomy, achievable with at least a 64-detector row scanner, allowing sub-millimeter spatial resolution [20]. This model is the foundation for all subsequent simulations and must faithfully capture vessel geometry. The importance of high-quality images lies in the fact that the estimated resistance of a coronary artery segment is calculated by applying the Poiseuille’s law, which describes resistance as inversely proportional to the fourth power of the vessel diameter. Therefore, limited spatial resolution may result in significant errors in the measurement of stenosis diameter, leading to exponentially greater inaccuracies in the assessment of stenosis resistance, pressure drop, and the calculated FFR-CT value [21]. FFR-CT analysis is enabled by automated image segmentation techniques that incorporate subvoxel resolution methods, in which individual voxels are subdivided into smaller elements with varying intensities based on the characteristics of adjacent subvoxels, in order to increase spatial resolution. CFD then requires the coronary lumen volume to be represented as a finite set of three-dimensional volumes (tetrahedral mesh elements).

The second step (quantification of the total and vessel resting coronary flow) involves calculating the myocardial mass using allometric scaling laws, which relate biologic variables to organism size. More specifically, under resting conditions, total coronary flow is directly proportional to myocardial mass, as expressed by $Q_{c}{\alpha M}_{\mathrm{myo}}^{0.75}$ ($Q_{c}$: coronary flow; $M_{\mathrm{myo}}$: myocardial mass; ^0.75^: scaling exponent). In this equation, myocardial mass can be accurately derived from CCT data [22].

The third step accounts for the calculation of myocardial microcirculatory resistance, by combining myocardial mass with an assumed mean aortic pressure. From the resulting total basal coronary resistance, outlet-specific resistance is estimated assuming resting resistance is inversely proportional to coronary diameter [23].

In the fourth step, maximal hyperemia is modeled by reducing microvascular resistance to ~24% of its resting level, a value derived from invasive measurements during intravenous adenosine infusion [24]. Notably, a ~4% coefficient of variation in patients with normal coronary flow reserve was registered, confirming consistent hyperemic response across individuals [25].

The last step applies Navier–Stokes equations derived from fundamental physics. These partial differential equations embody conservation of mass and momentum:

(1) $$\frac{\partial u}{\partial t}+(u\cdot \nabla )u=-\frac{1}{\rho }\nabla p+\nu \nabla^{2}u$$

This equation represents a fluid dynamics implementation of Newton’s second law (F=ma), where u is velocity, p is pressure, ρ is density, and ν is kinematic viscosity. In large coronary vessels (>500 μm), blood approximates Newtonian fluid with constant viscosity (~3–4 cP). Solving these equations yields spatial distributions of velocity and pressure along the coronary tree by using three-dimensional models of blood flow. Finally, FFR-CT, at any point distal to a stenosis, is then calculated as the ratio of simulated distal coronary pressure to proximal (aortic) pressure under modeled hyperemic conditions. This computational approach underpins CFD-based FFR-CT, requiring substantial processing power to solve millions of interdependent equations throughout the coronary tree. Consequently, CCT data are securely transferred to HeartFlow core laboratory where off-site supercomputers complete the analysis in 1–4 h. The report, containing personalized three-dimensional modeling and estimated point-by-point FFR-CT values on the depicted coronary tree, are sent back to the ordering clinician within 48 h.

From a technical standpoint, current bottlenecks of CFD-based FFR-CT lie in image quality dependency, segmentation errors, modeling assumptions, computational cost and processing time. Image quality, in terms of noise, inadequate contrast opacification or motion artifacts, directly affects the accuracy of lumen segmentation, which represents the geometric basis for FFR-CT computation. For example, motion artifacts or insufficient contrast opacification may cause overestimation of lumen size, potentially leading to falsely elevated FFR-CT values, or branch omission, which biases CFD boundary conditions. Conversely, severe coronary calcifications may lead to blooming artifacts and systematic underestimation of lumen diameter, resulting in artificially increased resistance and lower computed FFR-CT values. These geometric inaccuracies propagate through CFD modeling, which, in turn, relies on assumptions, such as fixed or predictable microvascular resistance, simplified blood flow physics, neglect of collateral coronary flow and the use of population-specific rather than patient-specific boundary conditions. Taken together, this may reflect downstream on diagnostic classification, particularly in lesions with borderline functional significance. Finally, the computationally intensive nature of these calculations may result in longer turnaround times. The main solutions are advances in image acquisition (motion correction algorithms, iterative reconstruction techniques, artificial intelligence-based image denoising, and photon-counting technology), improved segmentation algorithms (artificial intelligence-powered segmentation), refined physiological modeling, computational acceleration strategies (cloud-based infrastructure), and hybrid machine learning–CFD frameworks. As a result, the HeartFlow algorithm is a continuously evolving product.

## 3. Diagnostic Accuracy

FFR-CT accuracy critically depends on the precision of the three-dimensional coronary geometry model. Although no specialized protocols are mandated for FFR-CT, adherence to Society of Cardiovascular Computed Tomography guidelines optimizes results by the liberal use of nitrates and beta-blockers, maximizing lumen visualization and minimizing motion artifacts [25].

The diagnostic performance of FFR-CT has been evaluated in various prospective, multicenter studies using invasive FFR as the reference standard and blinded core lab assessment. FFR-CT has demonstrated robust diagnostic accuracy for identifying hemodynamically significant coronary stenoses (invasive FFR ≤ 0.80), consistently outperforming CCT alone through enhanced specificity and reduced false positives. The DISCOVER-FLOW trial evaluated FFR-CT performance using a first-generation algorithm in 103 patients (159 vessels) with suspected or known CAD. Invasive FFR ≤ 0.80 and CCT stenosis ≥ 50% defined ischemia. FFR-CT achieved superior accuracy (84% vs. 59%) and specificity (82% vs. 40%) versus CCT, with comparable sensitivity (88% vs. 91%). ROC-AUC favored FFR-CT (0.90 vs. 0.75, *p* = 0.001), with strong invasive FFR correlation (r = 0.717, *p* < 0.001) and minimal bias [26]. A strong correlation of FFR-CT with angiography-based quantitative flow ratio has also been shown [27].

Similar results emerged from the DeFACTO study (n = 252), which showed improved diagnostic performance (73% vs. 64% for CCT alone) and sensitivity for ischemia detection (90% vs. 84%), although specificity remained modest (54%). More importantly, in patients with intermediate stenoses, FFR-CT demonstrated substantially improved sensitivity (82% vs. 37%) [28]. The NXT trial (n = 254 patients, 607 vessels) provided definitive superiority versus CCT in the largest cohort: per-patient AUC 0.90 (95% CI 0.87–0.94) vs. 0.81 (0.76–0.87, *p* = 0.0008). FFR-CT demonstrated markedly higher specificity with comparable sensitivity, correctly reclassifying 68% of CCT false positives [29]. The NXT trial possessed key limitations. Notably, 68% of participants exhibited normal invasive FFR and exclusion of patients with high BMI and established CAD potentially introduced selection bias, limiting applicability to these high-risk subgroups.

FFR-CT was compared against CCT, 99m-Tc-tetrofosmin SPECT and 15O-H2O PET in a sub-analysis of the PACIFIC trial (n = 208 patients with suspected CAD). Per-vessel and per-patient AUC favored FFR-CT (0.94) over CCT (0.83), SPECT (0.70), and PET (0.87). However, intention-to-diagnose analysis favored PET due to FFR-CT non-evaluability because of artifacts [30], highlighting FFR-CT image quality dependency. Further evidence was provided by The SYNTAX III REVOLUTION trial which investigated whether CCT with FFR-CT could be used instead of ICA to guide Heart Team decisions about revascularization strategy (coronary artery bypass grafting [CABG] or PCI) in patients with complex CAD (left main and/or three-vessel disease). The agreement between modalities was very high and FFR-CT data influenced decision-making, changing treatment plans in around 7% of patients and modifying which vessels were targeted in 12% of cases compared with anatomy alone [31].

A recent meta-analysis of 43 studies (5236 patients, >7000 vessels) offers the most comprehensive assessment of FFR-CT diagnostic performance versus invasive FFR. Pooled diagnostic metrics demonstrated strong performance: accuracy 82.2%, sensitivity 80.9%, specificity 83.1%. Diagnostic certainty was excellent at spectrum extremes: FFR-CT value >0.90 ruled out significant CAD in 93% of cases, while a value <0.49 identified ischemia with approximately 100% accuracy. Intermediate “gray zone” values (0.75–0.80) showed reduced reliability, often necessitating further testing [32].

FFR-CT diagnostic performance has also been compared to CCT perfusion, an alternative method for assessing the functional significance of CAD, in the PERFECTION study. FFR-CT showed no significant differences in terms of diagnostic performance as compared to CCT perfusion and both provided additional diagnostic value on top of CCT alone in symptomatic intermediate or high-risk patients undergoing ICA with FFR measurement [33,34,35]. With respect to CCT perfusion, FFR-CT does not require additional contrast injections and radiation exposure; however, it is currently unfeasible in patients that have undergone coronary revascularization.

### Specific Subsets of Patients

The clinical yield of FFR-CT has been confirmed in various subsets of patients, including those older than 65 years and individuals with diabetes, both categories where obstructive disease is more prevalent [36,37]; however, its diagnostic accuracy depends critically on high-quality CCT and is influenced by CCT-specific image-degrading factors that may reduce reliability [38]. Extensive coronary artery calcification somehow impairs the diagnostic performance of both FFR-CT and CCT. A meta-analysis of 10 studies demonstrated superiority of the former over the latter for examinations with coronary artery calcium scores < 400, with higher per-patient/per-vessel AUCs. CFD-based modeling mitigates calcification artifacts affecting CCT lumen assessment; however, severe calcification (coronary artery calcium scores ≥ 400) reduces both specificity and AUC, warranting caution in FFR-CT interpretation in this subset of patients [39]. However, there is currently no universally accepted absolute threshold at which FFR-CT should be systematically avoided. Instead, most evidence supports a case-by-case approach, taking into account overall image quality, distribution and severity of calcification, and clinical pre-test probability. In selected patients with high calcium burden, FFR-CT may still provide useful incremental information if image quality is sufficient for reliable reconstruction, whereas alternative functional assessment strategies may be more appropriate when severe calcification is expected to preclude accurate analysis.

Another clinical setting where FFR-CT may prove useful is the assessment of acute chest pain patients presenting to the emergency department. Initial studies showed feasibility in this population and demonstrated association of abnormal FFR-CT values with acute coronary syndrome and revascularization [40]. However, a larger study of 555 patients did not find differences in clinical outcomes between a CCT-only and a CCT+FFR-CT approach in the emergency department [41]. Nonetheless, negative FFR-CT was associated with excellent long-term prognosis [42]. In a prospective study of higher-risk patients presenting with non-ST segment elevation acute coronary syndrome, FFR-CT demonstrated superior, although not statistically significant, diagnostic performance and greater capability to exclude hemodynamically significant stenoses compared with CCT alone, suggesting that FFR-CT may help limit unnecessary ICA by more precisely identifying individuals who require further intervention [43].

**Figure 1:**
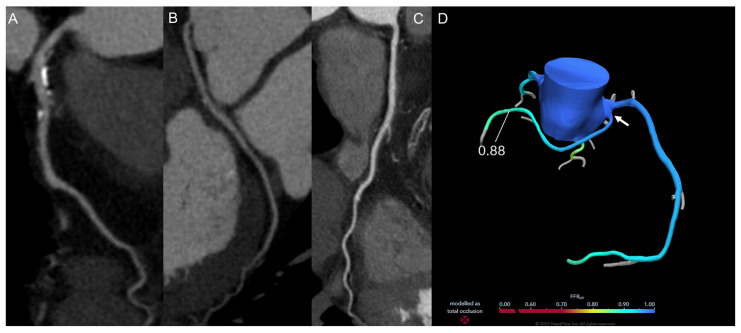
A 75-year-old woman, a former smoker, who had dyslipidemia, hypertension, and mild carotid artery disease, presented with effort dyspnea and atypical chest pain. CCT showed mild left main and left anterior descending artery disease (**A**) as well as an anomalous origin from the right coronary sinus and retro-aortic course of the left circumflex artery (**B**). The right coronary artery was free from disease (**C**). FFR-CT assessment was within normal range, including in the left circumflex artery (arrow; (**D**)). The patient was medically managed with no adverse events at follow-up.

FFR-CT has also demonstrated high diagnostic accuracy in evaluating anomalous aortic origin of coronary arteries (Figure 1), where anatomical imaging may fail to elucidate functional significance. In the ANOCOR registry (n = 62), FFR-CT values were significantly reduced in anomalous coronary arteries with high-risk morphological features of intramural course (take-off angle < 30°, luminal narrowing > 50%, eccentricity > 1.5). A proximal cut-off ≤ 0.83 achieved 96% sensitivity and 100% specificity for intramural pathway detection [44]. Notably, this cut-off is different from the conventional one used for atherosclerotic CAD.

FFR-CT has also been increasingly applied to evaluate microvascular dysfunction, the pathophysiological basis of angina/ischemia with non-obstructive coronary arteries. Specifically, the HeartFlow software may be used to calculate the coronary volume-to-myocardial mass (V/M) ratio; low values indicate potential microvascular angina or supply–demand mismatch, revealing ischemia causes despite minimal luminal stenosis [45]. In a prospective study (n = 153), 68 patients exhibited coronary microvascular dysfunction: these patients showed markedly reduced coronary flow reserve versus controls (1.9 ± 0.38 vs. 3.2 ± 0.81; *p* < 0.001), with strong inverse correlation between CCT-derived lumen volume and microvascular resistance (r = −0.59; 95% CI: −0.71 to −0.45; *p* < 0.001) and 40% smaller epicardial lumen volumes (512.8 ± 130.3 vs. 853.2 ± 341.2 mm; *p* < 0.001). Lumen volume independently predicted high resistance [46]. Thus, FFR-CT with V/M analysis may allow non-invasive assessment for subjects with angina/ischemia with non-obstructive coronary arteries.

Despite women typically exhibiting smaller epicardial coronary vessels than men, FFR-CT demonstrates equivalent diagnostic accuracy and discriminatory ability for ischemia detection across sexes (AUC: 0.93 vs. 0.90; *p* = 0.43), reducing non-obstructive CAD results at ICA and enhancing CCT revascularization prediction without sex-based differences [47]. To investigate potential implications for women, who historically undergo revascularization less frequently, a post hoc analysis from the ADVANCE registry was undertaken: women exhibited lower rates of obstructive CAD on CCT, higher FFR-CT values, and a reduced probability of FFR-CT ≤0.80 for the same degree of stenosis. Although referral rates to ICA following a positive FFR-CT were similar between sexes, women more often had non-obstructive findings at angiography and underwent revascularization less frequently, except when FFR-CT was ≤0.75, at which point revascularization rates converged with those of men [48].

## 4. Prognostic Value of FFR-CT

For several years, the long-term prognostic utility of FFR-CT and its incremental value in risk stratification beyond anatomical findings from CCT remained uncertain and required further prospective validation [21]. However, over the past decade a growing body of evidence from large-scale multicenter observational studies and post hoc registry analyses with follow-up of up to five years has consistently underscored the prognostic value of FFR-CT [49]. One of the earliest signals supporting this concept emerged from the PROMISE FFR-CT sub-study, where a FFR-CT value ≤ 0.80 proved to be a markedly stronger predictor of revascularization or major adverse cardiovascular events compared with severe stenosis on CCT (HR 4.3; 95% CI 2.4–8.9 vs. HR 2.9; 95% CI 1.8–5.1; *p* = 0.033) [50]. These findings were subsequently corroborated in the ADVANCE registry, which enrolled 5083 patients with suspected CAD and >30% stenosis on CCT. In this real-world international cohort, a reduced FFR-CT ≤ 0.80 was associated with a significantly higher one-year risk of cardiovascular death or myocardial infarction (RR 4.22; 95% CI 1.3–14; *p* = 0.01), although the interpretation of outcomes must be viewed in light of intrinsic limitations of registry-based research, including referral bias and the absence of precise lesion-level physiological co-localization [51].

Long-term observations from the Danish ADVANCE registry further reinforced these results, demonstrating that an abnormal FFR-CT was associated with an increased risk of all-cause death and spontaneous myocardial infarction over three years. Importantly, this prognostic association persisted even in patients with high coronary artery calcium scores (≥400), highlighting the robustness of FFR-CT for risk stratification across a broad spectrum of CAD severity [52]. Complementary evidence was provided by a meta-analysis including more than 5000 patients, where FFR-CT ≤ 0.80 was associated with a greater risk of all-cause mortality or myocardial infarction at 12-month follow-up, as well as a higher risk of major adverse cardiovascular events, all-cause mortality, myocardial infarction or unplanned revascularization. Notably, a clear risk continuum was observed, with each 0.10-unit decrease in FFR-CT corresponding to an increased likelihood of adverse events (RR 1.67; 95% CI 1.47–1.87; *p* < 0.001) [53].

Despite the inclusion of large and representative real-world populations, this body of evidence remains constrained by the observational design of most contributing studies, with inherent referral bias and confounding by indication, as downstream management was neither randomized nor standardized and may have been influenced by FFR-CT findings. Additional limitations include low absolute event rates, the absence of individual-level adjusted analyses, incomplete reporting of event timing, medical therapy and angina status, and restricted generalizability due to the predominantly low-risk population enrolled.

More recent real-world data have explored the prognostic implications of FFR-CT not only for diagnostic assessment but also for treatment decision-making. In a large observational registry comprising 7541 patients, including 1601 with suspected obstructive CAD, incorporation of FFR-CT findings into heart team decision pathways was associated with improved post-revascularization outcomes. Among the 559 patients undergoing revascularization (69.0% PCI, 29.7% CABG, 1.2% hybrid), 252 (45.1%) were evaluated using FFR-CT. Over a mean follow-up of 4.4 ± 2.2 years, 137 patients (24.5%) experienced major adverse cardiovascular events, with FFR-CT use associated with a favorable trend toward risk reduction and a significant decrease in all-cause mortality [54]. Taken together, these findings support the concept that FFR-CT-guided physiological stratification refines patient selection for revascularization and may translate into improved long-term outcomes. However, a recent meta-analysis challenged the role of FFR-CT in patients with stable chest pain, confirming that its use was associated with lower rates of ICA, but showing higher rates of coronary revascularization and no difference in terms of adverse events at follow-up [55].

Additional long-term prognostic evidence derives from studies directly comparing FFR-CT with invasive FFR. In one investigation with a mean follow-up of 9.9 years, an FFR-CT threshold of 0.80 independently predicted target vessel failure (HR 2.61; 95% CI 1.06–6.45). Among vessels in which revascularization was deferred, higher FFR-CT values were associated with lower risk (HR 0.62 per 0.1 increase; 95% CI 0.44–0.86), with cumulative incidences of target vessel failure of 2.6%, 15.2% and 28.6% for FFR-CT values > 0.90, 0.81–0.90 and <0.80, respectively (*p* for trend = 0.005). Importantly, FFR-CT demonstrated prognostic discrimination comparable to invasive FFR (c-index 0.79 vs. 0.71; *p* = 0.28) [56].

Collectively, these converging data position FFR-CT as a powerful prognostic marker which, akin to invasive FFR, should be interpreted as a continuous physiological variable rather than a dichotomous threshold. Nonetheless, the predominance of retrospective registries and post hoc analyses highlights the need for future prospective studies with longer follow-up to definitively establish its incremental prognostic role and optimal integration into clinical decision pathways.

## 5. Implementation of FFR-CT into Clinical Practice

FFR-CT application has become a key component of contemporary non-invasive coronary assessment, extending the role of CCT from purely anatomical stenosis evaluation to a combined anatomical and functional characterization of CAD. While invasive FFR continues to represent the reference standard for lesion-specific physiological assessment [57], its broader adoption is limited by procedural invasiveness, resource burden and the higher cost associated with pressure-wire-based assessment. In this context, FFR-CT provides a clinically meaningful non-invasive alternative that links anatomical stenosis severity with hemodynamic relevance, thereby improving patient selection for ICA and refining revascularization decision-making [58]. Recently, an international consensus document has been published encouraging the standardized, evidence-based application of FFR-CT in clinical practice [59].

**Figure 2:**
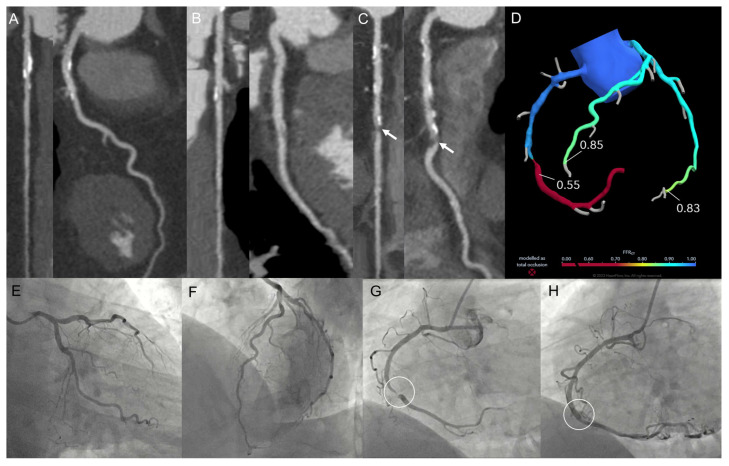
A 60-year-old man, former smoker, with dyslipidemia and a family history of CAD, presented with an onset of effort chest pain. CCT demonstrated mild stenoses at left anterior descending (**A**) and left circumflex arteries (**B**) and a severe stenosis at mid-right coronary artery (arrows; (**C**)). Pathological FFR-CT assessment at mid-right coronary artery; normal FFR-CT values at distal left circumflex and left anterior descending arteries (**D**). ICA confirmed non-obstructive disease of the left circumflex and left anterior descending arteries (**E**,**F**) and severe stenosis of the mid-right coronary artery (white circle; (**G**)). The patient was treated with percutaneous coronary intervention and drug-eluting stent implantation (white circle; (**H**)).

The added value of FFR-CT is greater in patients with coronary anatomy that is considered intermediate-risk: one or more lesions producing 30–69% luminal narrowing, or severe (≥70%) stenosis in coronary arteries other than the left main or proximal left anterior descending artery. In this setting, the functional assessment provided by FFR-CT helps guide decision-making between proceeding to ICA or continuing with optimal medical therapy alone (Figure 2) [60].

In contrast, FFR-CT is usually unnecessary in low-risk anatomy, such as in individuals with a normal CCT study or stenosis < 30%, where optimal medical therapy alone is appropriate. It is also not routinely recommended in high-risk anatomy, including in cases of ≥50% left main disease, severe proximal left anterior descending stenosis, or three-vessel disease. In these high-risk scenarios, direct referral for ICA is generally favored because the probability of hemodynamically significant coronary disease is already substantial.

The present clinical deployment of FFR-CT application is closely aligned with major international guideline recommendations. The 2021 ACC/AHA Chest Pain Guidelines assign FFR-CT a Class IIa recommendation for further functional evaluation of 40–90% stenoses identified on CCT in patients presenting to the hospital with acute or stable chest pain, supporting its role as an appropriate non-invasive adjunct to guide referral to invasive testing [61]. Within the European Society of Cardiology Guidelines for Chronic Coronary Syndromes, FFR-CT is recognized as a selective second-line modality to refine functional assessment following abnormal or inconclusive CCTA, particularly where disease is intermediate-grade or anatomically extensive; its use is recommended in adequately equipped centers and positioned as complementary to established non-invasive functional imaging and to invasive functional assessment during coronary angiography [4].

### Implementation of FFR-CT in Specific Contexts

**Figure 3:**
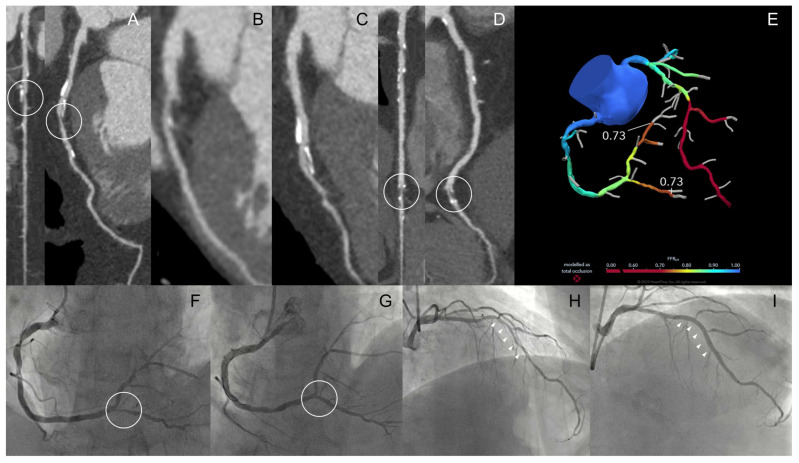
A 63-year-old man, smoker, known for diabetes, hypertension and dyslipidemia presented with effort chest pain (walking), resolved at rest. CCT showed severe stenosis of the left anterior descending artery (white circles; (**A**)), mild disease of the first (**B**) and second (**C**) diagonal branches and severe stenosis of the right coronary artery-posterior descending artery (white circles; (**D**)). FFR-CT confirmed the functional significance of both severe stenoses (**E**). The patient underwent percutaneous coronary intervention and drug-eluting stent implantation on the right coronary artery-posterior descending artery (white circles, (**F**,**G**)) and left anterior descending artery (white arrowheads; (**H**,**I**)).

In parallel with evidential maturation, FFR-CT application has expanded into more complex anatomical and procedural contexts [62]. In multivessel disease and chronic total occlusions, CCT already contributes to risk stratification and procedural planning. FFR-CT provides lesion-specific physiological assessment supporting more selective and tailored revascularization strategies in case of multivessel disease or serial/diffuse lesions (Figure 3).

Its utility has also been demonstrated in structural heart disease pathways. In the CT2TAVI study, a combined CCT + FFR-CT protocol implemented during pre-procedural transcatheter aortic valve implantation planning reduced the need for routine ICA and avoided unnecessary invasive testing in 60.5% of cases in which CCT revealed obstructive or non-interpretable proximal segments, while one-year mortality, myocardial infarction and revascularization outcomes remained comparable to standard care [63]. This constitutes the first prospective real-world study to introduce routine CCT ± FFR-CT for coronary assessment prior to transcatheter aortic valve implantation, reinforcing the feasibility and safety of CCT-based triage in high-risk elderly populations. In the FUTURE-AS registry, CCT with nitroglycerin and selective beta-blockers use was safe, highly evaluable (99.7%), and demonstrated 100% per-patient sensitivity and negative predictive value for detecting significant CAD (≥50%), while FFR-CT improved specificity and positive predictive value. Using a strategy consisting of deferring ICA in patients with <50% or ≥50% stenosis with FFR-CT >0.75 could potentially avoid ICA in 81.7% of patients, streamlining pre-transcatheter aortic valve implantation assessment [64].

A further domain of application has emerged within CCT-guided surgical revascularization strategies. The FASTTRACK-CABG program evaluated the feasibility of planning and performing CABG based exclusively on CCT and FFR-CT, withholding conventional ICA unless clinically necessary [65]. Within this setting, the introduction of the quantitative left-ventricular myocardial blood flow distribution percentage (LV%MYO) metric enabled objective, patient-specific estimation of myocardium at risk before surgery and quantification of residual myocardium at risk following revascularization. Among 106 analyzable patients, pre-CABG global LV%MYO was 70.1 ± 18.8%, decreasing to 14.0 ± 15.3% post-CABG in the 96 patients with follow-up imaging. Complete revascularization, defined as residual LV%MYO ≤ 10%, was achieved in 43.8% of cases [66]. These results illustrate how FFR-CT application contributes not only to lesion-level functional assessment, but also to fully CCT-guided surgical revascularization strategies.

FFR-CT technology also enables “virtual stenting,” using a planning tool in which a coronary stenosis is digitally modified to approximate post-percutaneous coronary intervention geometry. Using the same patient-specific three-dimensional coronary model and CFD framework, the stenotic segment is virtually restored to a reference lumen diameter, and pressure and flow are recalculated under simulated hyperemic conditions. This allows estimation of the predicted post-intervention FFR without performing an invasive procedure [67]. The Heartflow Planner demonstrated high accuracy in predicting FFR after stenting, even in diffuse and calcific disease [68,69]. The Precise Procedural and PCI Plan (P4) study (NCT05253677) is an ongoing multicenter randomized controlled trial designed to evaluate the use of CCT+FFR-CT to guide PCI. 

In addition to its role in revascularization planning, growing interest has focused on the interaction between FFR-CT estimates and coronary plaque characteristics derived from CCT. Emerging evidence indicates that plaque volume, composition, presence of high-risk features, and spatial distribution influence hemodynamic behavior and functional lesion significance [57,70,71]. The integration of plaque characterization with physiological indices represents an evolving dimension of FFR-CT application, with potential implications for risk stratification, monitoring of disease progression and preventive therapeutic decision-making within comprehensive CCT-based assessment frameworks.

**Figure 4:**
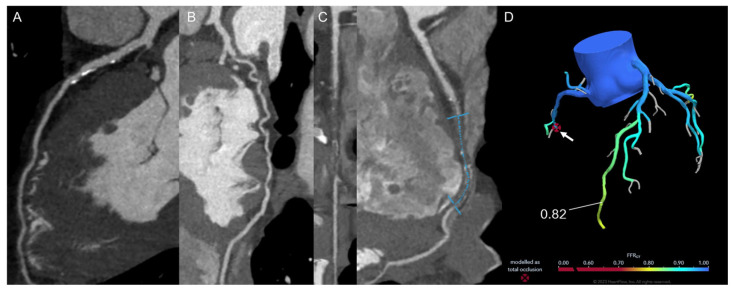
A 60-year-old man with family history of CAD, hypertension and dyslipidemia complained of recent onset of effort dyspnea. CCT showed non-obstructive disease of the left anterior descending artery (**A**) and left circumflex artery (**B**), and a long (50 mm) chronic total occlusion of mid-right coronary artery (**C**) with collateral supply from the left circumflex artery. FFR-CT showed absent functional relevance of the other stenosis and correctly displayed the occlusion (**D**). The patient underwent percutaneous recanalization of the right coronary artery with multiple drug-eluting stents implantation with symptom resolution.

Taken together, the cumulative evidence demonstrates that FFR-CT application enhances diagnostic discrimination, improves the appropriateness of ICA referral, increases the therapeutic yield of invasive procedures and supports patient-specific revascularization planning across multiple clinical contexts, including stable chest pain, multivessel coronary disease, pre-transcatheter aortic valve implantation assessment and CCT-guided CABG. However, its utility is limited in cases of chronic total occlusion. Because FFR-CT modeling assumes preserved antegrade coronary flow, complete occlusions fall outside the typical physiological conditions for which the technique was validated. Therefore, chronic total occlusions are not modeled, they are simply labeled as an X sign and Heartflow does not simulate collateral circulation (Figure 4).

## 6. Limitations of FFR-CT and Improvement Strategies

The performance of FFR-CT is critically dependent on high-quality CCT, as accurate lumen segmentation and myocardial mass estimation require optimal spatial resolution and full myocardial coverage [14,72]. Image-degrading factors including motion artifacts, calcium blooming, misalignment, excessive noise, inadequate contrast opacification or incomplete myocardial coverage may obscure lumen boundaries or exclude parts of the myocardium, leading to rejection of 3–25% of studies as unsuitable for analysis [29,73,74,75]. Notably, motion artifacts account for around 80% of excluded scans [50], and FFR-CT has not been validated in patients with coronary stents [76], bypass grafts, or following acute coronary syndrome, where metal artifacts, altered flow conditions and complex vessel remodeling may impair modeling accuracy. To minimize motion-related degradation, several best-practice acquisition strategies are recommended, including strict heart rate control (ideally <65–70 beats per minute) using beta-blockade when not contraindicated, administration of sublingual nitrates to optimize coronary dilation, and patient coaching to ensure consistent end-inspiratory breath-hold and avoidance of respiratory motion during image acquisition. In the emergency department setting, where patients with high or irregular heart rhythms are common, optimization of acquisition protocols to improve image quality is even more important, for example, using motion correction algorithms (e.g., SnapShot Freeze 2, GE HealthCare, Chicago, IL, USA), high-pitch “flash” acquisitions or dual-source systems, which intrinsically possess higher temporal resolutions. In patients with heavy coronary calcification, advances in image reconstruction techniques (iterative reconstruction models, such as ASiR-V, GE Healthcare, or SAFIRE/ADMIRE, Siemens Healthineers, Erlangen, Germany, and deep learning image reconstruction models), along with improved segmentation algorithms, may reduce blooming artifacts and enhance lumen delineation. For post-stent evaluation, approaches including photon-counting technology and machine learning-assisted analysis show promise, although further validation is required.

FFR-CT depends on precise anatomical and physiological modeling. Errors in the modeling process can lead to subsequent inaccuracies in FFR-CT measurements and their interpretation. For this reason, it is recommended to compare FFR-CT results with the original coronary CCT images and the corresponding anatomical FFR-CT model, especially when the values appear inconsistent [77].

The cost of FFR-CT and reimbursement issues represent an important consideration for its wider adoption. Evidence from the PLATFORM study demonstrated that the incorporation of FFR-CT in the evaluation of stable patients with chest pain was associated with a 32% reduction in costs in the invasive testing cohort ($7343 vs. $10,734) and with improved quality of life in the non-invasive group compared with usual care [78]. Similarly, in the FORECAST trial (n = 1400), an FFR-CT-guided strategy did not significantly alter total costs compared with standard care, but it significantly reduced the rate of ICA (19% vs. 25%; *p* = 0.01), supporting its potential role in limiting unnecessary invasive procedures [79]. Overall, these findings suggest that FFR-CT has the potential to improve resource utilization, particularly by optimizing patient selection for invasive testing, which, in turn, carries additional costs. However, more recent analyses have reported heterogeneous economic outcomes, reflecting differences in healthcare systems, reimbursement models, and real-world implementation settings [80], with some studies suggesting more limited or context-dependent economic benefit [81,82]. While the per-analysis costs of FFR-CT remain potential barriers, particularly in settings with limited reimbursement, economic modeling studies suggest that long-term cost-effectiveness may be achievable through reductions in unnecessary downstream testing and improved patient triage. Taken together, current evidence supports a potentially favorable but context-dependent economic profile, highlighting the need for further real-world validation across different healthcare environments [57]. Finally, the currently available evidence is derived from studies evaluating off-site FFR-CT analysis, such as HeartFlow. However, there is a lack of data assessing the economic performance of emerging on-site solutions, and their cost-effectiveness compared with off-site models remains to be evaluated.

## 7. FFR-CT Interpretation

In this section practical information for interpreting FFR-CT values will be provided. The FFR-CT report contains a three-dimensional model that allows the assessment of FFR-CT at each point across the coronary tree for vessels > 1.8 mm in diameter [49]. Smaller vessels that are not modeled are colored in gray. To assess for lesion-specific ischemia, FFR-CT should be measured 1–2 cm distal to the stenosis, differently from validation studies, where measurement location was matched with invasive FFR wire positioning.

The diagnostic accuracy of FFR-CT depends heavily on where the measurement is taken. To assess lesion-specific ischemia most reliably, values should be obtained 1–2 cm beyond the stenosis rather than at the distal end of the vessel, where a physiologic pressure drop exists, even without disease, which may falsely exaggerate the degree of ischemia [83]. A study comparing the accuracy of lesion-specific FFR-CT (measured 2 cm distal to the stenosis) versus distal-vessel FFR-CT in predicting the need for revascularization demonstrated that 44% of positive distal FFR-CT values were reclassified as negative when assessed lesion-specifically, avoiding overestimation of ischemia, and revascularization was more frequent in patients with positive lesion-specific FFR-CT values [84].

**Figure 5:**
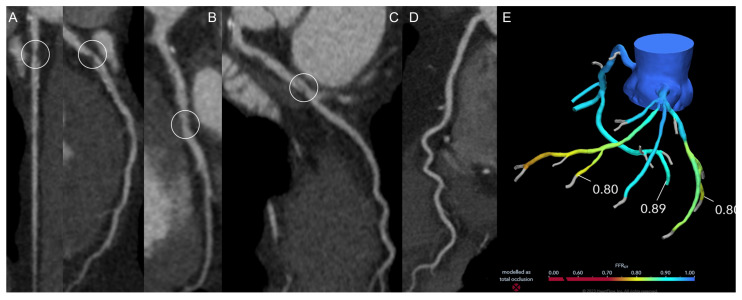
A 54-year-old male, with hypertension and dyslipidemia, presented for atypical chest pain onset (retrosternal dull chest pain with onset not reproducible at a definite threshold). CCT demonstrated moderate disease at proximal left anterior descending artery (white circles; (**A**)) and left circumflex artery (white circles; (**B**,**C**)). The right coronary artery had only minimal disease (**D**). FFR-CT was negative (**E**) and the patient was treated conservatively.

If the lesion-specific FFR-CT value is >0.80, the lesion is considered without functional relevance and unlikely to benefit from revascularization (Figure 5).

Conversely, if the FFR-CT value distal to the stenosis in question is lesser than or equal to 0.75, the lesion is categorized as positive by FFR-CT or hemodynamically significant. In addition to binary diagnostic performance, accumulating data indicate that the clinical interpretation of FFR-CT is intrinsically value-dependent [85]. The “gray zone” of FFR-CT values between 0.75 and 0.80 represents a diagnostic conundrum, as lesions in this range are functionally significant at invasive FFR in about half of cases [86]. In case borderline values are obtained, clinical decisions should be individualized based on patent symptoms, ΔFFR-CT values, lesion characteristics such as location (left anterior descending artery vs. other vessels) and segment (proximal vs. distal), number of affected vessels, total plaque burden, and presence of high-risk plaque features (e.g., low-attenuation plaque, napkin-ring sign, and positive remodeling), using a stepwise interpretative approach [77]. In the absence of high-risk features or symptoms, many patients in this range may be safely managed with medical therapy and close clinical follow-up, with ICA reserved for persistent or worsening symptoms.

Sometimes a discordance between CCT and FFR-CT is observed. This is not unexpected, as the relationship between anatomic stenosis severity and physiologic impact is not linear. At CCT, approximately 21% of patients with mild luminal narrowing in the 30–49% range demonstrate abnormal FFR-CT results; conversely, about 28% of patients with severe stenosis (70–90%) may still have normal FFR-CT values [87].

Beyond relying solely on absolute FFR-CT cut-off values, ΔFFR-CT offers additional functional information by quantifying the pressure drop across a coronary stenosis. This is especially valuable in functionally intermediate lesions, where the presence and extent of ischemia may be uncertain. Importantly, ΔFFR-CT has emerged as a clinically meaningful parameter for differentiating focal from diffuse coronary artery disease and for informing decisions between revascularization and optimal medical therapy [88]. From a practical point of view the stenotic segment is first assessed on the three-dimensional FFR-CT reconstruction. Reference points are then selected in vessel segments free of visible luminal disease 1–2 cm upstream and downstream of the narrowing. ΔFFR-CT is subsequently determined by calculating the difference between the FFR-CT values obtained at these two locations. In a study, a ∆FFR-CT ≥0.12 strongly predicted hemodynamic significance at invasive FFR (odds ratio 10.2) [89], with best diagnostic accuracy (AUC 0.86), as compared to other non-invasive measurements. Additionally, the prognostic role of ΔFFR-CT in predicting adverse events in patients with non-obstructive CAD has been shown [90,91]. While this approach enables lesion-specific assessment of pressure gradients, it introduces a degree of operator dependency. Variability in the exact placement of these reference points may lead to inter-observer differences in ΔFFR-CT values, especially in cases of diffuse disease or serial stenoses where lesion boundaries are less clearly defined.

## 8. Alternative Methods for Deriving FFR-CT

Despite the HeartFlow off-site solution being the most validated approach, economic considerations, reimbursement variability, the need for remote data transfer and longer processing times, as mentioned above, limit its widespread availability. These limitations as well as advancements in artificial intelligence-based solutions are driving the growing development of on-site FFR-CT technologies that could theoretically overcome some of these concerns.

Current on-site and emerging artificial intelligence-based approaches can be broadly grouped into three categories: (i) physics-based or CFD-derived models and their machine learning surrogates, (ii) hybrid physics–machine learning models incorporating physiological constraints, and (iii) purely data-driven, imaging- or plaque-feature-based models that infer ischemia without explicit flow simulation.

In the first category, traditional CFD models solve Navier–Stokes equations on patient-specific coronary geometries reconstructed from CCT. These models are typically coupled with assumptions regarding boundary conditions to compute pressure drops and derive FFR-CT values. To reduce computational burden, several deep learning surrogate models have been developed. These typically employ convolutional neural networks, graph neural networks, or encoder-decors architectures (e.g., U-net-based variants) trained to approximate CFD-derived pressure fields or directly predict FFR values from coronary geometry [92,93,94,95]. However, these models remain sensitive to distribution shifts across scanner and institutions, and their internal decision pathways are largely non-interpretable, limiting the mechanistic traceability of predictions.

Hybrid approaches attempt to combine physiological modeling with data-driven learning. These include physics-informed neural networks, which embed governing fluid dynamic constraints into the loss function, as well as models that integrate anatomical descriptors (e.g., vessel curvature, stenosis geometry) with learned representations from imaging data. Architecturally, these systems often combine convolutional feature extractors with constraint-based optimization layers or differentiable fluid dynamics modules. By explicitly encoding physiological priors, they aim to improve generalizability compared with purely data-driven models [96,97,98]. The main advantage of these models lies in improved physiological consistency and potential robustness in anatomically complex cases. However, they are computationally more complex than purely data-driven approaches and remain less mature in terms of clinical validation and regulatory approval.

The third category includes purely data-driven approaches that infer ischemia without explicit hemodynamic simulation. These systems rely on machine learning classifiers or deep neural networks trained on imaging-derived features, including plaque composition, stenosis severity, perivascular fat attenuation, and global coronary burden. Architecturally, these methods typically use gradient-boosted decision trees, random forests, or deep multilayer perceptrons, as well as convolutional neural network-based feature extraction pipelines followed by classification heads. Unlike CFD-based methods, they do not explicitly model coronary flow but instead learn statistical associations between imaging phenotypes and invasive FFR-defined ischemia [99]. A key limitation of this category is reduced physiological interpretability, as predictions are driven by learned correlations rather than explicit hemodynamic modeling. Conversely, these approaches offer advantages in speed, scalability, and ease of deployment, particularly in fully automated pipelines.

Across these three categories, a clear trade-off emerges between physiological interpretability, computational complexity, and scalability. CFD-based models provide strong mechanistic grounding but are computationally intensive and dependent on multiple modeling assumptions. Hybrid approaches attempt to balance physiological realism with data-driven flexibility but remain methodologically complex and still require extensive validation. Pure data-driven models offer the greatest scalability and workflow integration potential but sacrifice explicit hemodynamic interpretability. Importantly, although current FFR-CT on-site platforms pursue comparable diagnostic objectives, heterogeneity in study design, datasets, and reference standards limits direct quantitative comparison across methods. Therefore, the present review focuses on conceptual and clinical implications rather than performance benchmarking, emphasizing how methodological choices influence interpretability, robustness, and potential clinical translation (Table 1).

From a financial prospective, because some of these solutions can run on standard local workstations, they usually require an initial investment for acquisition of the software package and clinician training for performing the analyses; however, once implemented, the evaluation of subsequent cases generally does not incur additional per-case costs, with faster result generation and lower computational demands.

Several studies investigated the diagnostic value of machine learning FFR-CT for the assessment of the hemodynamic relevance of coronary stenoses, including in patients with prior coronary stenting, who cannot be assessed with CFD-based FFR-CT [100,101,102]. A recent meta-analysis assessed the diagnostic performance of machine learning-based FFR-CT compared with CFD-based FFR-CT. It included 23 studies covering 2501 patients and 3764 vessels in the CFD cohort, and 1323 patients with 4194 vessels in the machine learning cohort. The findings showed similar specificity and AUC between the two techniques at both the patient and vessel levels. The diagnostic odds ratio at the vessel level was likewise comparable. However, machine learning FFR-CT demonstrated significantly lower sensitivity than CFD-based FFR-CT at both patient and vessel levels [103]. The currently observed lower sensitivity may be attributed to several factors: quality, size, and representativeness of the training datasets, inability to fully capture the underlying physiological constraints in complex anatomies, or variability in imaging protocols, scanner types, and preprocessing pipelines across institutions.

Several commercially or experimentally available platforms illustrate these approaches. One of such technologies is a prototype research software (xFFR, GE HealthCare) that allows for on-site deep learning and fluid dynamic-based derivation of FFR values [104]. A study by Fazzari et al. prospectively compared xFFR to off-site FFR-CT and invasive FFR, showing that xFFR is a robust and efficient on-site tool for assessing CAD demonstrating high diagnostic accuracy and reproducibility. Notably, xFFR was compared to off-site FFR-CT and showed better results, particularly in more difficult cases such as overweight patients, mixed plaques, and less-than-ideal image quality. Interestingly, although both modalities demonstrated strong agreement relative to invasive FFR, they show only a moderate level of agreement when compared directly with each other [105]. Its rapid processing (around eight minutes) and integration into clinical workflows position xFFR as a promising alternative to off-site FFR-CT solutions [106]. In another study, a good diagnostic performance against invasive instantaneous wave-free ratio was also shown [107]. 

uCT-FFR (United Imaging Healthcare, Shanghai, China) is an on-site CFD-based algorithm using alternative boundary conditions based on transluminal attenuation gradients and delivering results in about eleven minutes, that has been validated against invasive FFR and demonstrated high accuracy in detecting functionally significant stenoses [108].

Cleerly (Cleerly Inc., New York, NY, USA), an AI-based platform that uses proprietary machine learning algorithms to quantify and characterize atherosclerotic plaque, coronary stenosis, and the likelihood of ischemia. Unlike CFD-derived FFR-CT approaches, it does not explicitly model coronary hemodynamics but instead infers ischemia probability from imaging-derived plaque and anatomical features. AI-QCT_ISCHEMIA_ is a Cleerly tool that delivers a binary result, classifying ischemia as either unlikely or likely, using a cut-off aligned with invasive FFR values (>0.80 vs. ≤0.80) [109]. In recent studies, its diagnostic accuracy was superior compared to off-site FFR-CT as well as other myocardial perfusion imaging techniques [99], and a positive result was associated with adverse outcomes [110,111].

## 9. Future Directions

On-site machine learning-based FFR-CT holds promise to substantially streamline clinical workflows by delivering results directly within the imaging facility, thereby reducing turnaround times and potentially improving overall cost-effectiveness. As noted, such platforms are designed to provide near real-time or minutes-scale functional assessment directly from CCT data, in contrast with the current off-site CFD-based pipeline. However, for newer FFR-CT platforms, prospective multicenter outcome studies and rigorous cost-utility analyses remain scarce. This evidence gap underscores the necessity for well-designed, multi-vendor comparative trials to more clearly establish long-term clinical impact, economic sustainability, and overall value within diverse healthcare settings.

Photon-counting computed tomography, a novel technology that uses photon-counting detectors instead of conventional energy-integrating detectors, has been recently introduced in clinical practice, with improved spatial resolution, contrast-to-noise ratio, and reduced blooming artifacts, with the promise to further improve diagnostic accuracy thanks to more precise lumen segmentation, more accurate stenosis quantification and more reliable flow modeling. Initial studies provide evidence of the feasibility of FFR-CT integration with photon-counting technology [112,113].

Another promising direction is the use of FFR-CT to study functional changes in coronary physiology in patients with CAD undergoing medical management with intensive lipid lowering therapies [114]. In the THRONE study, for instance, most intermediate coronary lesions initially classified as FFR-CT-negative tended to worsen over time, with nearly 50% becoming hemodynamically significant within two years by FFR-CT [115].

## 10. Conclusions

In conclusion, FFR-CT is a technique derived from standard CCT datasets without the requirement of additional imaging or radiation exposure and represents a significant advancement in non-invasive cardiovascular imaging by combining anatomical information from CCT with functional assessment of coronary lesions. It offers the potential to improve diagnostic accuracy, guide patient management, and reduce unnecessary invasive procedures, acting as a gatekeeper for the catheterization laboratory. While clinical studies have demonstrated its reliability and prognostic value, ongoing research is needed to optimize its integration into routine practice, address cost-effectiveness, and expand its applicability across diverse patient populations. Unlike stress-based functional tests, which primarily detect flow-limiting ischemia, CCT with FFR-CT can also identify and characterize non-obstructive CAD. This is clinically important because non-obstructive atherosclerosis carries independent prognostic significance, even in the absence of ischemia. By simultaneously evaluating plaque burden and composition, lesion-specific ischemia, and overall coronary anatomy, novel machine learning FFR-CT tools may provide a more comprehensive evaluation of coronary risk and improve risk stratification compared with functional or anatomical testing alone. The increasing availability of diverse FFR-CT methodologies may ultimately benefit both patients and healthcare systems by improving accessibility, reducing costs, and enabling more flexible integration into clinical workflows. Overall, FFR-CT holds promise as a transformative tool in personalized, evidence-based management of CAD.

## Figures and Tables

**Table 1:** Overview of methodological approaches for non-invasive estimation of fractional flow reserve from coronary computed tomography angiography (FFR-CT). Abbreviations: AI = artificial intelligence; CCT = cardiac computed tomography; CFD = computational fluid dynamics; CNN = convolutional neural network; FFR-CT = fractional flow reserve derived from computed tomography; ML = machine learning; PINNs = physics-informed neural networks; U-Net = convolutional neural network architecture for image segmentation.

Approach Category	Traditional CFD-Based FFR-CT (e.g., Heartflow)	CFD Surrogate Models (AI-Assisted)	Physics-Informed ML Models	Plaque-Feature-based Models (e.g., Cleerly)
Methodological basis	Physics-based fluid dynamics (Navier–Stokes equations)	Data-driven approximation of CFD outputs	Hybrid physics + learning constraints	Probabilistic ischemia inference
Typical algorithms/architectures	Numerical solvers, finite element/finite volume methods	CNNs, U-Net variants, graph neural networks	PINNs, physics-constrained neural networks	Proprietary ML pipelines
CFD dependency	High	Low (trained on CFD outputs)	Partial	None
Input data	Coronary geometry from CCT + boundary conditions	Coronary geometry from CCT	CCT + physiological constraints	Coronary plaque + morphology features
Interpretability	High (physiological basis explicit)	Low–moderate	Moderate	Moderate
Computational demand	High	Low–moderate	Moderate	Very low
Validation level	Extensive (clinical validation studies)	Moderate (often internal validation)	Limited–moderate	Moderate (prognostic data available)
Clinical readiness	Established (off-site commercial systems)	Emerging	Early research	Clinically emerging

## Data Availability

No new data were created or analyzed in this study. Data sharing is not applicable to this article.

## Abbreviations

CABG	Coronary artery bypass grafting
CAD	Coronary artery disease
CCT	Cardiac computed tomography
CFD	Computational fluid dynamics
FFR	Fractional flow reserve
FFR-CT	Fractional flow reserve derived from coronary computed tomography angiography
ICA	Invasive coronary angiography
LV%MYO	Left-ventricular myocardial blood flow distribution percentage
PCI	Percutaneous coronary intervention
